# Release of Tongue-Tie in Neonates

**Published:** 2012-01-01

**Authors:** V Raveenthiran

**Affiliations:** Department of Pediatric Surgery, SRM Medical College Hospital, SRM University Kattankulathur, Chennai, India

## INTRODUCTION

Arguably, release of tongue tie is the oldest surgical procedure and it is perhaps older than circumcision. According to the Old Testament, the Lord released the tied tongue of Moses and made him speak well [1]. Tongue-tie is pedantically known as ankyloglossia inferior minor. Simplicity of its treatment has earned this malady several etiological attributions such as difficulty of speech and breast feeding. All over the world, lactation consultants refer neonates for release of tongue tie as they believe it to be the cause of maternal nipple pain.Perhaps this is the only indication of tongue tie release in neonates.

As early as 1473 CE Bartholomaeus Metlinger suggested that midwifes should grasp the tongue of a newborn with honey-smeared fingers and tear the frenulum with the sharp nails [1]. Interestingly, there is a sharp divide among various specialists regarding the influence of ankyloglossia on breast feeding. About 99% of lactation consultants believed that tongue tie is the cause of neonatal feeding difficulties while only 30% of otolaryngologists and 10% of pediatricians did so [2]. The controversy is further complicated by the fact that neonates with ankyloglossia did not significantly differ from newborn with normal tongue as regard to the success of breast feeding (83% Vs 92% respectively) and the incidence of maternal nipple pain (22% Vs 20% respectively) [3] These facts inevitably cause reverberation of a two-century-old quote of the Berlin obstetrician Hagen.

“Among ten infants, in whom the midwife or the nurse seek thereason for poor sucking in a short frenulum, barely one has this cause ... Frequently the parents are deceived, for profit, greed and ignorance this aid is abused, and one unties where nothing is tied” [1].

Science tends to neglect problems that are simple to solve. Accordingly, the literature on tongue tie is devoid of robust science; but is full of dogmatic, distortive and delusory data. High quality scientific evidence such as well conducted large randomized controlled trials (RCT) and meta-analyses are conspicuously missing or scarce [4,5]. In this background, a recently published article of Buryk et al [6] - which appears to be a seminal contribution on this subject - has been considered herein for detailed dissection and critical analysis.

## THE STUDY

The study is published in the August 2011 issue of Pediatrics [6]. The journal is prestigious because it is the official organ of American Academy of Pediatrics and its impact factor of 5.39 (the highest among pediatric journals). The study has been published from Naval Medical Center Portsmouth, Virginia. The lead author is a pediatrician and all the authors have disclosed no conflicts of interest. The study design had been approved by Institutional review board of the authors’ center.

This is a single-blind RCT involving 58 newborns who met enrollment criteria. The mean age at enrollment was 6 days (SD 6.9; range 1-35). The total number of newborns delivered during the study period was 3025. Notably, none of those who met inclusion criteria refused to participate in the study and there were none who met exclusion criteria. Mothers experiencing nipple pain or breast feeding problem were referred to a lactation consultant who examined the neonates’ tongue and graded the degree of tongue-tie using ‘Hazelbaker Assessment Tool for Lingual Frenulum Function’ (HATLFF). A functional score more than 11 or an appearance score less than 8 were set as indications of frenotomy. The 58 neonates who met this criteria were randomized into sham group (n=28) and frenotomy group (n=30). Mothers were blinded as to the nature of intervention; but this blinding is short-lived only up to the time of first feeding after the intervention, as mothers could not contain their curiosity to look into the infant’s mouth. Frenotomy was done without any anesthesia by crushing the frenulum with a hemostat and cutting it with a sharp scissors. Contrary to this, the sham group neonates were retained in the procedure room for a specified time and returned to mothers. Maternal nipple pain and ability to breast feed were assessed by Short-Form McGill Pain Questionnaire (SF-MPQ) and Infant Breastfeeding Assessment Tool (IBFAT) respectively. Neonates were breast fed immediately after the procedure. Maternal scores, before and after the intervention, are compared to assess the effectiveness of frenotomy.

Both the frenotomy and control group showed statistically significant drop in maternal pain scores. However, the frenotomy group showed significantly more improvement than the sham group. IBFAT scores showed statistically significant improvement in frenotomy group than the control group. All these differences persisted only for the first 2 to 3 weeks of the intervention and there was no significant difference between the groups beyond the third week. Many mothers of the sham group subsequently opted for frenotomy. Thus crossing over of groups prevented any long term assessment beyond 4 weeks. The authors conclude that frenotomy causes immediate improvement in nipple pain and breastfeeding scores, despite a placebo effect on nipple pain.

## DISSECTIONS

This study, seemingly of sound science, has several pitfalls. First of all it is a single blind study. Mothers, but not the observers, were blinded. Thus, an observer bias is not excluded. Some of the assessment tools used such as HATLFF have lot of scope for observer bias. For example, in HATLFF elasticity of frenulum is graded as 0, 1 and 2 when frenular elasticity is little, moderate and excellent respectively. It is anybody’s guess as to how an observer will determine between two adjacent qualitative measures such as ‘little’ and ‘moderate’. Inter-rater variations of these tools have been acknowledged by authors themselves. Ideally, the study should have been a double blind trial. It is best known to authors as to why they have not designed it as double-blind especially when there are no ethical or financial implications of it.

Secondly, scoring tools used in this study are of questionable reliability and perhaps they appear absurd on several counts. For example, in HATLFF tool “lifting of tongue” is graded according to the degree of lift. How is it possible to command or stimulate a newborn to lift its tongue to the maximum possible extent? Similarly peristalsis of tongue is graded as complete, partial or none. It is perplexing as to how the authors could observe and grade peristalsis which occurs during sucking within closed mouth. HATLFF was developed by Alison Hazelbaker for her Master’s degree thesis in 1993 [7]. Soon, it was adopted by many without critical external validation. A recent study from Minnesota University found this tool useless in identifying tongue-tied infants at risk of breast feeding problems [7]. Despite this scientific evidence, the authors went ahead to choose this questionable tool for defining inclusion criteria. Thus, a selection bias could not be ruled out.

Thirdly, SF-MPQ tool used for scoring maternal nipple pain also appears irrational. In fact, it was not originally designed to score nipple pain and it has several regional modifications. It scores 15 different characters of pain such as “Throbbing, Shooting, Stabbing, Sharp, Cramping etc”. Each of these pain descriptions are subjectively graded 0 to 3 when they are none to severe respectively. In addition to this, pain is evaluated 0 to 5 when it is none to excruciating. Thus, a total score of 50 determines the degree of nipple pain in SF-MPQ tool. Several of these pain descriptions overlap each other. Further, how is it possible for anyone to experience all these characters of pain in varying quantities? Even if they can, what is the scientific significance of various characters of pain? Thus, the very design of SF-MPQ tool itself looks illogical. Similarly, validity and reliability of IBFAT tool has been questioned by several studies [8,9]. Usage of these disputed tools undermines the reliability of the study by introducing the bias of inappropriate tools.

Fourthly, it is not clear as to how the authors applied nipple pain score. Ideally, it should be applied separately for each breast of an individual, both before and after the experimental intervention. The paired data thus generated should then be evaluated for intervention effect. Authors have not followed this rigorous scientific path in their study design. This may potentially introduce contamination bias. For example, nipple sore need not be symmetrical in a given individual. If the authors had applied pre-intervention scoring on the side of sore nipple and post-intervention scoring on the contralateral nipple of the same individual, the results ought to show spurious improvement.

Fifthly, performing frenotomy in the experimental group without any anesthesia could possibly be unethical. Authors might have influenced IRB to do away with anesthesia as it may unmask blinding in the experimental group. Despite this precaution, the blinding methodology could still be imperfect. It may not be impossible for an intuitive mother to suspect frenotomy from the painful cry of neonates of the experimental group as compared to calm newborns of sham group. Further, the blinding is only ultra short-lived until the first feeding. Lack of long-term evaluation is certainly the biggest demerit of the study. The issue is made murkier by the crossing over of the two groups when most of the mothers of the sham group opted for frenotomy within first few weeks of the study.

Sixthly, there can be many differential diagnoses for nipple pain in a breast feeding mother. Amir has listed as many as 35 causes [10]. Tongue tie is only one among the 35 causes. A frequent cause of breast pain during lactation is bacterial or candida mastitis. According to an Australian survey [11], nearly 15 to 20% mothers suffer mastitis mostly within the first 4 weeks of breastfeeding. Mastitis is often subclinical and is frequently associated with nipple damage. Authors of the paper under discussion made no effort to exclude other causes of maternal nipple pain. In fact, they did not even attempt lactation counseling. There are no data as to how many of these mothers were primigravidas and multigravidas. Obviously, the first-time mother may experience nipple pain due to incorrect feeding technique. Inclusion of all those who has pain with an assumption that tongue tie as the root cause, introduces ‘preformed opinion’ bias.

Lastly, the temporary improvement observed in frenotomy group can be explained by several confounding factors. The two groups showed improvement in pain and breast-feeding score in the first three weeks of the study and subsequently the groups did not differ. Interestingly, both the groups showed a significant drop in pain score thereby confirming a placebo effect or a maternal adaptation response. It is well known that a newborn learns to properly latch after the first week of life [12]. Correlating this fact with the mean age of 6 days reported in the study, one may easily suspect that the problem could be due to improper technique rather than tongue tie. Mothers who are naïve to breast feeding could have learnt the proper technique and art by the two weeks’ time. Neonates of the frenotomy group might be sucking less vigorously due to surgical pain than the control group, thereby accounting for the reduced maternal pain scores. Other causes of nipple pain such as subclinical mastitis might have resolved by the end of second week thereby causing illusionary improvement in scores. Lack of control over these issues introduces the bias of confounding factors.

## DISCUSSION

The very concept of subjecting a newborn for a painful procedure for the sake of alleviating mother’s distress is against ethical principles. How can John be robbed to benefit Tom? If the prime aim is to relieve the nipple pain of mother, it is surprising as to why everybody indulges in cutting the neonate’s frenulum rather than trying topical anesthetics for the mother. Could it be due to conflicting interest of financial incentives? Why potent topical anesthetics such as EMLA (Eulatic mixture of local anesthetics) have not been given a trial?

Attributing tongue tie as the cause of poor latch and nipple pain is conceptually illogical. The misconception is rooted on physician ignorance on neonatal anatomy. Short lingual frenulum is physiological in newborn as is non-retractable prepuce in this age group [12]. Further, it is perplexing as to how a soft, lubricated tongue of neonates can damage maternal nipple and cause pain.

## CONCLUSION

The pitfalls of the study are improper single-blinding design, scoring tools of doubtful validity, contamination of collected data, lack of control over confounding factors, lack of long term data, crossing over of study groups, preformed opinion bias and potential selection bias. Many of the pitfalls are quite serious to the extent of invalidating the conclusions of the study. Having been published in a prestigious journal, this study is now part of literature. According to the rungs of evidence based medicine, this study provides level-1 (highest) published evidence in favour of neonatal frenotomy. But a critical dissection has exposed several serious pitfalls in study design and analysis. What should a practicing neonatal surgeon infer from this study? Should the he /she change the practice based on this study or not? Perhaps, Peter Morgan’s poetry [13] answers these moot questions better:

“………… Even if an RCT says yea or nay, The doctor has the right to throw the book away If his or her opinion strongly disagrees With the massive RCT and its plethora of ‘p’s” - Peter Morgan. [13].

## Footnotes

**Source of Support:** Nil

**Conflict of Interest:**The author of the critical review personally believes that tongue tie is extremely rare and very seldom causes breast feeding problems. His belief could have subconsciously influenced the selection of this paper for critical analysis and opinions expressed therein.

